# Association between vitamin D receptor gene polymorphisms (Fok1 and Bsm1) and osteoporosis: a systematic review

**DOI:** 10.1186/s40200-014-0098-x

**Published:** 2014-10-17

**Authors:** Zahra Mohammadi, Fateme Fayyazbakhsh, Mehdi Ebrahimi, Mahsa M Amoli, Patricia Khashayar, Mahboubeh Dini, Reza Nezam Zadeh, Abbasali Keshtkar, Hamid Reza Barikani

**Affiliations:** Department of biology, Damghan branch, Islamic Azad University, Damghan, Iran; Osteoporosis Research Center, Endocrinology and Metabolism Clinical Sciences Institute, Tehran University of Medical Sciences, Tehran, Iran; Endocrinology and Metabolism Research Center, Endocrinology and Metabolism Clinical Sciences Institute, Tehran University of Medical Sciences, Tehran, Iran; Biomedical Engineering Department, Maziar University, Rouyan, Iran; Non-communicable Disease Department, Iran Ministry of Health and Medical Education, Tehran, Iran; Dental Implant Research Center, Tehran University of Medical Sciences, Tehran, Iran; EMRI, Dr Shariati Hospital, North Karegar St., Tehran, 14114 Iran

**Keywords:** Osteoporosis, Vitamin D receptor gene, Bone density, Polymorphism, Fok1, Bsm1

## Abstract

Osteoporosis is a health concern characterized by reduced bone mineral density (BMD) and increased risk of fragility fractures. Many studies have investigated the association between genetic variants and osteoporosis. Polymorphism and allelic variations in the vitamin D receptor gene (VDR) have been found to be associated with bone mineral density. However, many studies have not been able to find this association. Literature review was conducted in several databases, including MEDLINE/Pubmed, Scopus, EMBASE, Ebsco, Science Citation Index Expanded, Ovid, Google Scholar, Iran Medex, Magiran and Scientific Information Database (SID) for papers published between 2000 and 2013 describing the association between Fok1 and Bsm1 polymorphisms of the VDR gene and osteoporosis risk. The majority of the revealed papers were conducted on postmenopausal women. Also, more than 50% studies reported significant relation between Fok1, Bsm1 and osteoporosis. Larger and more rigorous analytical studies with consideration of gene-gene and gene-environment interactions are needed to further dissect the mechanisms by which VDR polymorphisms influence osteoporosis.

## Introduction

### The genetic variants of osteoporosis

Bone is a metabolically active tissue that experiences continuous remodeling via two reciprocal processes, bone formation and resorption. Respectively, osteoclasts, osteoblasts and osteocytes are responsible for bone resorption, formation and maintenance [1]. Osteoporosis is a bone disease characterized by low bone density caused by increased activity of osteoclasts and decreased bone turnover [2,3].

The prevalence of osteoporosis varies between different populations and ethnic groups [4-6] for example because of the high degree of ethnic variety in China, different studies show variety prevalence of osteoporosis [7-24]. Considering its high prevalence, the disease imposes a heavy burden on the patients and families as well as the healthcare system. In fact, the numbers of women with osteoporotic fractures are higher than those who experience breast, ovary and uterus cancer [25-27].

Osteoporosis is a disease caused by the interaction of genetic and environmental factors. According to many studies, the contribution of genetic and environmental factors is about 70% and 30% respectively. The environmental factors can control gene expression and accordingly, the process of the disease [28]. The study showed that 60-80% feature of bone mass depends on genetics. The Caucasians and Asians usually have lower bone density values than Negros, Hispanics and Latino Americans [29]. In addition, studies have shown that female offspring of osteoporotic women have lower bone density in comparison with that of those with normal bone density values [30]. Similarly, male offspring of men who are diagnosed with idiopathic osteoporosis have lower BMD in comparison with that of men with normal bone density values [31]. Also, the study of female twins have shown heritability of BMD to be 57% to 92% [32-35]. Different approaches including linkage studies on human and experimental animals as well as candidate gene studies and alterations in gene expression are being used currently to identify the role of genes in this regard [36].

There are many relevant published studies of the genetic susceptibility to osteoporosis. Genes can affect the skeletal system in two ways. The first, control body uptakes and intakes such as urinary calcium excretion to modulate BMD, the second way is poor metabolism due to genetic defects [37].

There calcium absorption pathways consists of trans_cellular and para_cellular. The trans_cellular pathway closely depends on the action of calcitriol and the intestinal vitamin D receptor. Transcellular transport occurs primarily in the duodenum where the VDR (Vitamin D receptor) is expressed in the highest concentration. So the regulation of VDR gene is most important in high efficiency of calcium absorption [38,39].

Estrogens are known to play an important role in regulating bone homeostasis and preventing postmenopausal bone loss. They act through binding to two different estrogen receptors (ERs), ERα and ERβ, which are members of the nuclear receptor superfamily of ligand-activated transcription factors. Both ER kinds are expressed in osteoblasts, osteoclasts, and bone marrow stromal cells. And also ESRα has a prominent role in regulating bone turnover and the maintenance of bone mass [40,41].

Different studies have reported a list of effective genes on osteoporosis; the most important of which are vitamin D receptor gene(VDR), estrogen receptor alpha (ESRα) ,interleukin -6 (IL-6), Collagen type I (COLIA1), LDL receptor-related protein 5 (LRP5) [26,42,43].

Over the recent decades, genome-wide association studies (GWAS) have contributed to the understanding of gene structure in complex and chronic diseases such as osteoporosis. Some of studies have indicated 62 significant loci where control bone mineral density variation [27,44-47].

#### Candidate genes for BMD

Candidate gene studies have mainly focused on Vitamin D receptor genes (VDR), type 1 Estrogen receptor genes (ESR1) and type 1 Collagen (Coli1) [41,48-50]. In this paper, the more important candidate genes, “VDR,” is discussed.

### Vitamin D receptor gene

Vitamin D receptor’s (VDR) genotypes have been associated with the development of several bone diseases as well as multiple sclerosis (MS), osteoporosis, and vitamin D-dependent rickets type II and other complex maladies [51].

The human gene encoding the VDR gene has been localized on chromosome 12q12-q14. Vitamin D receptors (VDRs) are members of the NR1I family, which also includes pregnane X (PXR) and constitutive androstane (CAR) receptors, which form heterodimers with members of the retinoid X receptor family [52]. VDR is expressed in the intestine, thyroid and kidney and has a vital role in calcium homeostasis. VDRs repress the expression of 1-alpha-hydroxylase (the proximal activator of 1,25(OH)2D3) and induce the expression of 1,25(OH)2D3 through inactivating the enzyme CYP24. Also, it has recently been identified as an additional bile acid receptor alongside FXR with a protective role in gut against the toxic and carcinogenic effects of these endobiotics [53].

Gene ontology (GO) annotations related to this gene include steroid hormone receptor activity and sequence-specific DNA binding transcription factor activity. An important paralog of this gene is NR4A3 [54].

There are more that 100 restriction endonuclease recognition sites in VDR gene and some of them are polymorphisms such as Fok1, Bsm1, Apa1 and …. .

Fok1 and Taq1 are located in exon 2 and 9 respectively. And also, Bsm1 and Apa1 are located in intron 8. Bsm1, Apa1 and Taq1 have been identified at the 3’ end of the gene. The effects of VDR gene polymorphisms are in connection with each other [55-57]. In many studies, polymorphisms of VDR gene have been investigated. A relationship between the VDR polymorphism and osteoporosis remain unclear requiring further in depth studies [58,59].

A series of characterized VDR gene polymorphisms, including Fok1, Bsm1, Taq1, and Apa1, have been extensively studied with regard to their association with osteoporosis, but with vise versa results [41,60-63].

Significant associations of Fok1 polymorphism with low BMD have been described in some studies, [64-66] but not in others [67,68].

The Bsm1 restriction enzyme identifies a polymorphic site at an intron at the 3′-end which is in linkage disequilibrium with several other polymorphisms, including Apa1, Taq1, and the variable-length poly(A) [69]. Although functional data have been inconclusive for Bsm1, several small studies evaluating Bsm1 have reported significant associations with osteoporosis [70,71].

To clear the relationship between osteoporosis and VDR gene polymorphisms (Fok1, Bsm1), we review the current evidence systematically.

## Methods

### Eligibility criteria

In this systematic review, the studies that had assessed the association between VDR gene polymorphisms and osteoporosis between 2000 and 2013 were included. In all these studies the diagnosis of osteoporosis was performed based on BMD measurement by Dual X-ray Densitometry (DXA) at least one of the bone sites. All kinds of original studies such as cross-sectional, longitudinal, and case controls were included. All review articles, Meta-analysis and systematic reviews after checking references (to avoid missing any paper), were excluded. Also, the articles performed on patients with secondary osteoporosis as well as non-human studies (cell culture or animal studies) and cellular-molecular discussions were excluded. To avoid language bias non-English-language publications were also included and Google-Translator was used to extract these articles’ data.

### Literature search and data extraction

The search strategy was based on electronic and hand searching. Main key words in this systematic review were Osteoporosis, Bone density, vitamin D receptor gene, polymorphisms, Fok1 and Bsm1. We searched electronic databases of biological and health sciences including MEDLINE (pubmed), Scopus, EMBASE, Ebsco, Science Citation Index Expanded, Ovid, Google Scholar, Iran Medex, Magiran and Scientific Information Database (SID). All national and international congresses about genetic and osteoporosis like IOF and NOF congresses were examined. And also expert’s curriculum vitae in this field were checked for relevant studies. Subsequently, the searches were carried out and publications of interest were selected, based on titles and abstracts. The full text of all selected publications was assessed for relevance. If the full texts of papers were not available, they were obtained through correspondence with the authors. This was followed by extracting the relevant data from the identified publications according to the steps described in detail below. Totally, two reviewers reviewed the articles. In case of disagreement, the third reviewer assessed the articles.

The following data were extracted from each published article: name of the first author, publication year, the number of case and control by gender, the number of menopausal women, and the number of performed BMDs, ethnic origin of the studied population, mean age, genotyping method (PCR-RFLP and TaqMan), Bone sites, and the genotype frequency of the polymorphisms. The reliability of data extraction forms was assessed by genetics and endocrinology specialists. And the content validity was assessed by 10 articles and was confirmed by 0.75 Cronbach’s Alpha. Methodological quality, the strength and weaknesses of included studies were investigated using a modified STROBE checklist.

## Results and discussion

### Baseline characteristics

**Figure 1:**
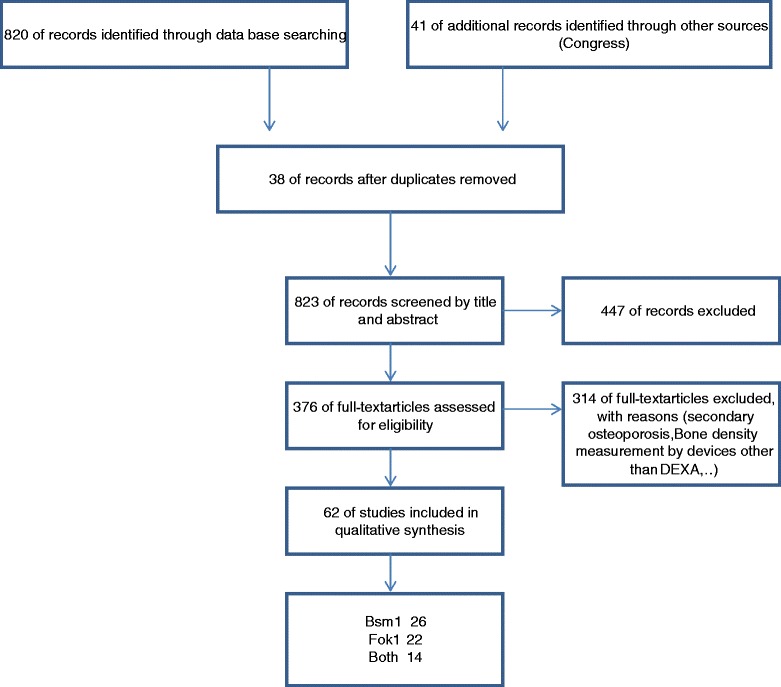
**Flow diagram of the literature search.**

A schematic of the literature search is shown in Figure 1. According to the inclusion/exclusion criteria eligibility, 61 articles were identified regarding the associations between the Fok1 and Bsm1 polymorphisms of VDR gene and osteoporosis risk. Among these studies, 21 studies concerned the association of the Fok1 polymorphism with osteoporosis [9,72-91], while 26 studies investigated the association between Bsm1polymorphism and osteoporosis risk [92-117]. Also 14 articles evaluated both polymorphism associations with osteoporosis [118-131]. All of these 61 studies provided sufficient data to calculate the possible relationship between the two polymorphisms of the VDR gene and osteoporosis risk. The general characteristics of the selected studies are summarized in Table 1.

**Table 1:** **Characteristics of studies included in the systematic review**

	**First author**	**Year**	**Country**	**Ethnicity**	**Genotyping method**	**Design**	**Total sample size**	**Osteoporosis**	**Control**	**SNPs**
1.	Wynne, F [72]	2002	Ireland	Irish	PCR-RFLP	Case-Control	511	381	130	**Fok1**
2.	Van Pottelbergh [73]	2002	Belgium	Belgium	PCR-RFLP	Case-Control	408	271	137	**Fok1**
3.	Tarner [74]	2012	Turkey	Turkish	PCR-RFLP	Case-Control	229	183	46	**Fok1**
4.	Jakubowska [75]	2012	Netherlands	Netherlands	PCR-RFLP	Case-Control	455	161	294	**Fok1**
5.	Kanan [76]	2013	Jordan	Jordanian	PCR-RFLP	Case-Control	210	120	90	**Fok1**
6.	Zhang [77]	2006	Chinese	Chinese	PCR-RFLP	Case-Control	92	26	66	**Fok1**
7.	Kim, J. G [78]	2001	Korea	Korean	PCR-RFLP	Cross-sectional	229	-	-	**Fok1**
8.	Deng, H. W [79]	2002	USA	Caucasian	PCR-RFLP	Cross-sectional	630	-	-	**Fok1**
9.	Lau, E. M. C [9]	2002	Chinese	Chinese	PCR-RFLP	Cross-sectional	684	-	-	**Fok1**
10.	Chen,H . Y [80]	2002	Taiwan	Taiwan	PCR-RFLP	Cross-sectional	163	-	-	**Fok1**
11.	Strandberg, S. [81]	2003	Sweden	Swedish	PCR-RFLP	Cross-sectional	88	-	-	**Fok1**
12.	Rabon-stith,K. M [82]	2005	USA	USA (Maryland)	PCR-RFLP	Cross-sectional	206	-	-	**Fok1**
13.	Cusack, S. [83]	2006	Denmark	Danish	PCR-RFLP	Cross-sectional	224	-	-	**Fok1**
14.	Terpstra [84]	2006	Netherlands	Netherlands	PCR-RFLP	Cross-sectional	120	-	-	**Fok1**
15.	Lau, H. H. L [85]	2006	Chinese	southern Chinese	PCR-RFLP	Cross-sectional	674	-	-	**Fok1**
16.	Falchetti, A. [86]	2007	Italy	Lampedusa (Italian)	PCR-RFLP	Cross-sectional	424	-	-	**Fok1**
17.	Han, X. [87]	2009	Chinese	Han	PCR-RFLP	Cross-sectional	100	-	-	**Fok1**
18.	Hosseinnejad [88]	2009	IRAN	IRAN	PCR-RFLP	Cross-sectional	205	-	-	**Fok1**
19.	Hosseinnejad [89]	2009	IRAN	IRAN	PCR-RFLP	Cross-sectional	312	-	-	**Fok1**
20.	Ozaydin [90]	2010	Turkey	Turkish	PCR-RFLP	Cross-sectional	88	-	-	**Fok1**
21.	Galbav [91]	2010	Slovakia	Slovak	PCR-RFLP	Cross-sectional	121	-	-	**Fok1**
22.	Perez, A. [92]	2008	Argentina	Cordoba	PCR-RFLP	Case-Control	176	108	68	**Bsm1**
23.	Fontova, R. [93]	2000	Spain	Spanish	PCR-RFLP	Case-Control	156	105	51	**Bsm1**
24.	Uysal, A, R. [94]	2008	Turkey	Turkish	PCR-RFLP	Case-Control	246	100	146	**Bsm1**
25.	Eckstein [95]	2002	Israeli	Jewish Israeli	PCR-RFLP	Case-Control	324	86	238	**Bsm1**
26.	Borjas-Fajardo. L [96]	2003	Spain	Spanish	PCR-RFLP	Case-Control	133	78	55	**Bsm1**
27.	DurusuTanriover, M. [97]	2010	Turkey	Turkish	PCR-RFLP	Case-Control	100	50	50	**Bsm1**
28.	Chen, J. [98]	2003	Chinese	Chinese	PCR-RFLP	Case-Control	61	40	21	**Bsm1**
29.	Tamulaitien [99]	2012	Lithuania	Lithuania	PCR-RFLP	Case-Control	73	28	45	**Bsm1**
30.	Nelson [100]	2000	USA	African-American	PCR-RFLP	Cross-sectional	43	-	-	**Bsm1**
31.	Sowinska [101]	2000	Poland	Polish	PCR-RFLP	Case-Control	88	40	48	**Bsm1**
32.	Chen,H.Y [102]	2001	Chinese	Chinese	PCR-RFLP	Cross-sectional	171	-	-	**Bsm1**
33.	Pollak, R. D [103]	2001	Israeli	Israelis	PCR-RFLP	Cross-sectional	634	-	-	**Bsm1**
34.	Kubota, M [104]	2001	Japan	Japanese	PCR-RFLP	Cross-sectional	126	-	-	**Bsm1**
35.	Laaksonen, M. [105]	2002	Finland	Finish	PCR-RFLP	Cross-sectional	93	-	-	**Bsm1**
36.	van der Sluis, I. M. [106]	2003	Netherlands	Caucasian	PCR-RFLP	Cross-sectional	148	-	-	**Bsm1**
37.	Grundberg [107]	2003	Sweden	Swedish	PCR-RFLP	Cross-sectional	343	-	-	**Bsm1**
38.	Kammerer, C. M. [108]	2004	Mexico	Mexican American	PCR-RFLP	Cross-sectional	471	-	-	**Bsm1**
39.	Seremak-Mrozikiewicz, A [132]	2004	Poland	Polish	PCR-RFLP	Cross-sectional	34	-	-	**Bsm1**
40.	Palomba, S. [110]	2005	Italy	Italian	PCR-RFLP	Cross-sectional	1100	-	-	**Bsm1**
41.	Dong, J. [111]	2006	Chinese	Han	PCR-RFLP	Cross-sectional	90	-	-	**Bsm1**
42.	Bernardes[112]	2005	Portugal	Portuguese	PCR-RFLP	Cross-sectional	114	-	-	**Bsm1**
43.	Mitra, S. [113]	2006	India	Indian	PCR-RFLP	Cross-sectional	246	-	-	**Bsm1**
44.	Bezerra [114]	2008	Brazil	Brazilian	PCR-RFLP	Cross-sectional	40	-	-	**Bsm1**
45.	Musumeci [115]	2009	Italy	Sicilian	PCR-RFLP	Cross-sectional	360	-	-	**Bsm1**
46.	Stathopoulou, M. G. [116]	2011	Greece	Greece	PCR-RFLP	Cross-sectional	578	-	-	**Bsm1**
47.	Pouresmaeili [117]	2013	IRAN	IRAN	PCR-RFLP	Cross-sectional	146	-	-	**Bsm1**
48.	Horst -Sikorska, W. [118]	2007	Poland	Polish	PCR-RFLP	Cross-sectional	279	-	-	**Both**
49.	Gonzalez [119]	2013	Mexico	Mexican-Mestizo	TaqMan	Case-Control	320	232	88	**Both**
50.	Kanan, R. M. [120]	2008	Jordan	Jordanian	PCR-RFLP	Case-Control	230	150	80	**Both**
51.	Lisker, R [121]	2003	Mexico	Mexican	PCR-RFLP	Case-Control	122	65	57	**Both**
52.	Mansour, L. [122]	2010	Egypt	Egyptian	PCR-RFLP	Case-Control	70	50	20	**Both**
53.	Rogers [123]	2000	no indicated	no indicated	PCR-RFLP	Cross-sectional	46			**Both**
54.	Lorentzon [124]	2001	Sweden	Caucasian	PCR-RFLP	Cross-sectional	99	-	-	**Both**
55.	Zajíčková [125]	2002	Czech	Czech	PCR-RFLP	Cross-sectional	114	-	-	**Both**
56.	Vidal, C. [126]	2003	Malta	Malta	PCR-RFLP	Cross-sectional	104	-	-	**Both**
57.	Bandrés [127]	2005	Spain	Caucasian	PCR-RFLP	Cross-sectional	177	-	-	**Both**
58.	Ivanova, J. [128]	2006	Bulgaria	Bulgarian	PCR-RFLP	Cross-sectional	219	-	-	**Both**
59.	Macdonald, H. M [129]	2006	UK	Scotland	PCR-RFLP	Cross-sectional	3100	-	-	**Both**
60.	Yavuz [130]	2007	Turkey	Turkish	PCR-RFLP	Case-Control	206	381	130	**Both**
61.	Sanwalka [131]	2013	India	Indian	PCR-RFLP	Case-Control	120	271	137	**Both**

In these studies, diverse groups of people were discussed. 36.5% of the studies studied postmenopausal women. On the other hand, post-menopausal and pre-menopausal woman were studied simultaneously in 22.2% of the articles and 12.7% of them studied all groups.

According to the results, 96.8% of studies performed the polymorphisms using PCR-RFLP “polymerase chain reaction- restriction fragment length polymorphism”. The other methods such as Taq-man were used to determine the association between Bsm1 and Fok1 polymorphisms and osteoporosis.

As mentioned above, the studies after year 2000 on world were enrolled in this systematic review. Most articles were published in 2006 and after that the number of papers showed a decline trend.

Based on articles, totally 17473 persons studied. 65.9% of studies reported a significant relation between Bsm1 and osteoporosis risk. Likewise, 60.0% of studies reported a significant relation between Fok1 and osteoporosis risk.

Also, the papers were categorized by gender and age. The data indicated that most of the articles were done on women and also on older ages. Most of studies were examined post menopausal women.

After characterization of authors” countries, it was demonstrated that respectively china [9,77,85,87,98,102] and Turkey [74,90,94,97,130] presented 6 and 5 studies and identified as most active countries in such researches.

In conclusion, both gender and ethnicity are effective factor on osteoporosis and bone mineral density.

As is evident, genetic variant has a tremendous roll to adjust bone activities and therefore along with vitamin D deficiency, has a large effect on osteoporosis incidence and also osteoporotic fractures. In this systematic review, due to study the association between low bone density and Bsm1 and Fok1 polymorphisms, 61 papers were studied and statistically analyzed. As a main result, most of the studies were performed on post-menopausal women i.e. the largest risk group to their major content of research [132]. It seems that it is necessary to evaluate the association of genetic variant for lower age groups of both genders. Accordingly, genetic testing can be used to prevent osteoporosis and low bone density.

In more than 50% of studies a significant association was found between the two polymorphisms (Fok1 and Bsm1) and osteoporosis. Based on the articles, 65.9% of studies reported a significant correlation between Bsm1 and osteoporosis risk. Likewise, 60.0% of studies reported a significant correlation between Fok1 and osteoporosis risk. Most of the studies were performed in developed countries but also, developing countries have initiated this way.

An important and noticeable issue in this systematic review is different results in different races [44,133]. Ethnicity and race, like gender, can influence the epidemiology of osteoporosis and BMD. Some of studies indicate that lowest BMD shown in white women and also, bone mineral density is higher in African Americans [134,135].

In more that 95% studies for assessing polymorphisms, PCR-RFLP were used (Table 1). It is noteworthy; Taq-Man is approximately novel methods which used [119].

## Conclusion

In summary, there is large ethnic and racial variability in BMD levels and osteoporosis rates. Across all racial groups and polymorphisms differences, women experience osteoporosis is more than the combined number of women who experience breast cancer. Prevention efforts should target all women, irrespective of their race and ethnicity, especially if they have multiple risk factors. And also, using novel and pioneer genetic techniques to better assess and better quality can be useful.
